# Host cell cytosolic immune response during *Plasmodium* liver stage development

**DOI:** 10.1093/femsre/fuy007

**Published:** 2018-02-26

**Authors:** Carolina Agop-Nersesian, Livia Niklaus, Rahel Wacker, Volker Theo Heussler

**Affiliations:** 1 Department of Molecular and Cell Biology, Henry M. Goldman School of Dental Medicine, Boston University, MA 02118, USA; 2 Institute of Cell Biology, University of Bern, Baltzerstrasse 4, CH-3012 Bern, Switzerland; 3 Graduate School for Cellular and Biomedical Sciences, University of Bern, CH-3012 Bern, Switzerland

**Keywords:** *Plasmodium*, autophagy, intracellular immune response, parasitophorous vacuolar membrane, PVM shedding, parasite evasion strategies

## Abstract

Recent years have witnessed a great gain in knowledge regarding parasite–host cell interactions during *Plasmodium* liver stage development. It is now an accepted fact that a large percentage of sporozoites invading hepatocytes fail to form infectious merozoites. There appears to be a delicate balance between parasite survival and elimination and we now start to understand why this is so. *Plasmodium* liver stage parasites replicate within the parasitophorous vacuole (PV), formed during invasion by invagination of the host cell plasma membrane. The main interface between the parasite and hepatocyte is the parasitophorous vacuole membrane (PVM) that surrounds the PV. Recently, it was shown that autophagy marker proteins decorate the PVM of *Plasmodium* liver stage parasites and eliminate a proportion of them by an autophagy-like mechanism. Successfully developing *Plasmodium berghei* parasites are initially also labeled but in the course of development, they are able to control this host defense mechanism by shedding PVM material into the tubovesicular network (TVN), an extension of the PVM that releases vesicles into the host cell cytoplasm. Better understanding of the molecular events at the PVM/TVN during parasite elimination could be the basis of new antimalarial measures.

## Teaser

Host hepatocytes recognize and attack invading *Plasmodium* parasites but the parasites have developed strategies to evade this response.

## INTRODUCTION

Host immune responses put a strong evolutionary pressure on pathogens including viruses, bacteria and parasites. With the evolvement of an intracellular lifestyle, many pathogens avoid direct confrontation with their host's humoral and cell-mediated immune responses. However, infected host cells are well equipped to fight intracellular invaders. Cellular homeostasis pathways and different cell death programs are engaged in diverse responses against intracellular pathogens. Since these responses are very variable and customized to different pathogen species, we combine them here under the term ‘intracellular immune responses’. Importantly, these defense pathways can proceed independently of specialized immune cells but are linked to the adaptive immune system by supporting major histocompatibility complex presentation of pathogen-derived peptides (Schmid, Pypaert and Münz 2007).

Elimination of intracellular pathogens can generally be distinguished either by host cell suicide responses where the pathogen is eliminated together with its host cell or by prosurvival responses that specifically recognize and lyse the pathogen without affecting the host cell. The fate of the infected host cell depends greatly on the response to inflammatory cytokines. Interferon (IFN)-signaling pathways, which trigger the activation of the inflammasome, induce a specialized programmed cell death, pyroptosis (see review Mitchell and Isberg 2017). Alternative host cell suicide programs, activated upon a microbial infection, involve apoptosis and necroptosis (reviewed in Jorgensen, Rayamajhi and Miao 2017). During *Plasmodium* liver infection, apoptosis (Van De Sand *et al.*^2005^; Leirião *et al.*^2005^; Leirião, Mota and Rodriguez 2005; Kaushansky *et al*. 2013a,b) and type I IFN-signaling pathways (Liehl *et al.*^2014^) represent host cell death-mediated defense mechanisms and have been discussed to contribute to the development of natural resistance to malaria. Sacrificing the infected host cell restricts dissemination of the pathogen and eventually sustains the integrity of the cell community. Some IFN-regulated GTPases, however, bypass the inflammasome signaling and contribute to the host cell survival either by restricting the pathogen growth or clearance of the pathogen. A central role in prosurvival signaling pathways play autophagy-associated responses, which can also be activated independent of an IFN response. Besides the polymerization of an actin cage around the *Plasmodium* parasite (Gomes-Santos *et al.*^2012^), autophagy and autophagy-related mechanisms have been recently described for *Plasmodium* infection of hepatocytes as important host defense strategy and will be the focus of this review.

Since the parasite resides within a parasitophorous vacuole (PV) during its entire liver stage development, the surrounding membrane serves as the main interface to the cytoplasm of the hepatocyte. Interestingly, this so-called parasitophorous vacuolar membrane (PVM) plays a fundamental role in the parasite's escape route from the host cell autophagic response. In this review, we will first briefly introduce the concept of autophagy followed by discussing molecular events during parasite invasion and the biology of the PVM before summarizing the main findings concerning intracellular host cell responses and then close with parasite evasion strategies and concluding remarks emphasizing the future challenges in this emerging field of research.

## FEATURES OF DIFFERENT AUTOPHAGY PATHWAYS

**Figure 1.**
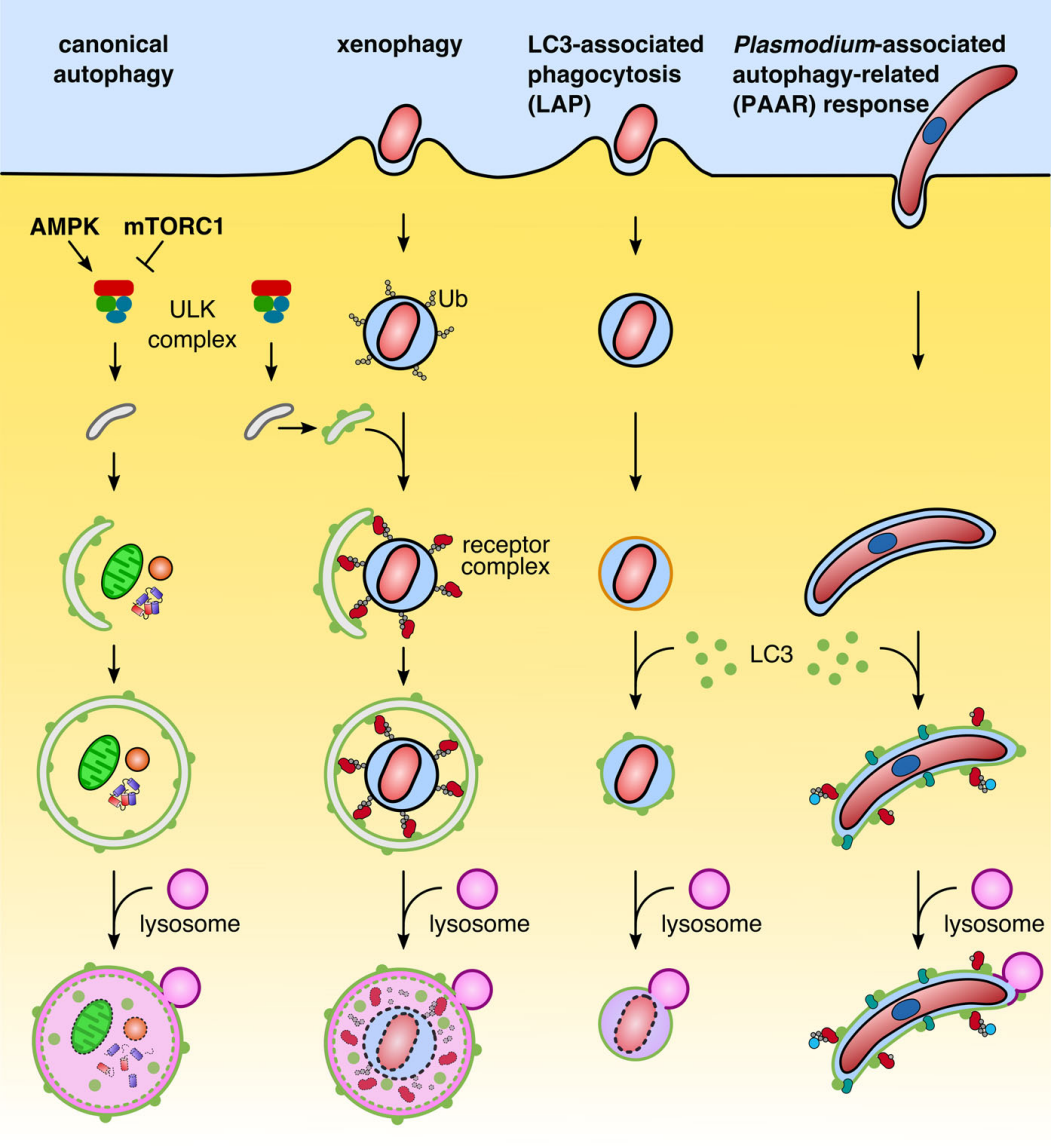
Overview of different autophagy pathways. Hallmark of canonical and selective autophagy—exemplified by xenophagy—is the formation of a double membrane autophagosome. Both pathways are characterized by a hierarchical sequence: initiation via the ULK complex, nucleation, elongation and closure, recycling and degradation by lysosomal enzymes. Canonical autophagy is activated by AMP-activated protein kinase (AMPK) and inhibited by mammalian target of rapamycin complex 1 (mTORC1). A distinctive feature of selective autophagy is the specific recognition of ubiquitinated cargo, which is recognized by autophagy receptors. The receptors mediate recruitment of the autophagosomal membrane. In LC3-associated phagocytosis (LAP), ATG proteins are directly recruited to a single-membrane vacuole. PI3P and the production of reactive oxygen species (ROS) define the target membrane for the LC3 lipidation machinery. The *Plasmodium*-associated autophagy-related (PAAR) response in *P.berghei* shares features of xenophagy and LAP. LC3 is directly incorporated into the parasitophorous vacuolar membrane (PVM) and recruits autophagy receptors and ubiquitin in an inverse order. Although *Plasmodium* has evolved strategies to avoid acidification of the parasitophorous vacuole (PV), parasites can be eliminated by the PAAR response of the host cell.

**Figure 2.**
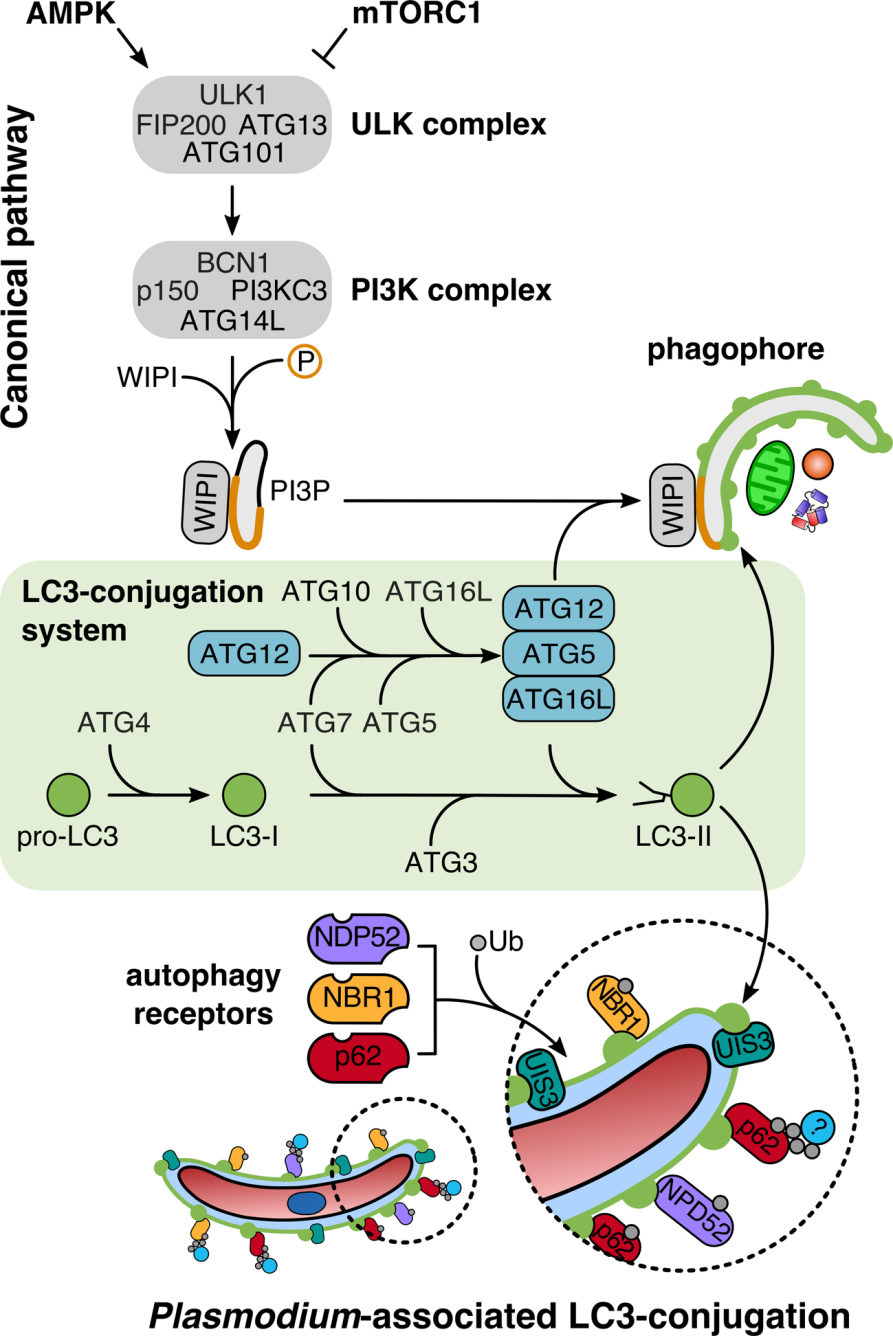
Comparison of the LC3-conjugation pathway during canonical autophagy and *Plasmodium*-associated autophagy-related (PAAR) response. Upper panel: Autophagosome formation during canonical autophagy depends on the multiprotein complex ULK and the class III phosphatidylinositol 3-kinase (PI3KC3) complex defining phagophore membranes with PI3P (orange). The lipid signal recruits the PI3P-dependent effector WIPI and the LC3-conjugation system to drive elongation of the LC3-positive phagophore (green). Both pathways share the LC3-conjugation system for LC3 lipidation (green box). Lower panel: PAAR response bypasses the canonical steps involved in the initiation, nucleation and elongation of the autophagosome. The pathway upstream of the LC3-conjugation system, which is important for the initiation of the response, and the recognition of the parasitophorous vacuolar membrane (PVM) is unknown.

To understand how intracellular pathogens interact with their host cells, it is important to distinguish starvation-induced canonical from pathogen-induced selective autophagy and other autophagy-related pathways (Fig. 1). All forms of autophagy share a core machinery as well as having pathway-specific components. In general, autophagy refers to a tightly regulated catabolic process that delivers cytoplasmic contents for lysosomal degradation. Both canonical and selective autophagy pathways are based on macroautophagy, which involves the formation of a double-membrane vesicle, the autophagosome. External and internal stimuli can enhance or mediate specific autophagy processes. Upon stress and nutrient deprivation, activation of canonical autophagy mediates bulk sequestration and self-digestion of parts of the cytoplasm and organelles. Two master regulators sense the metabolic status of the cell. The two antagonists, the activator AMP-activated protein kinase (AMPK) and the inhibitor mammalian target of rapamycin complex 1 (mTORC1), act as a molecular switch to control activity of the initiation complex ULK in a phosphorylation-dependent manner (Kim *et al.*^2011^) (Fig. 2). Activation of the ULK complex, an assembly of Unc51-like kinase 1 and 2 (ULK1/2), autophagy related protein (ATG) 13, focal adhesion kinase family interacting protein of 200 kD (FIP200) and ATG101, initiates the autophagy response. The enhanced kinase activity of ULK phosphorylates the class III phosphatidylinositol 3-kinase (PI3KC3) complex, resulting in local enrichment of phosphatidylinositol 3-phosphate (PI3P) (Russell *et al.*^2013^). This signaling lipid defines the pre-autophagosomal membrane (e.g. endoplasmatic reticulum, ER) and recruits downstream effector proteins such as WD repeat domain phosphoinositide-interacting proteins (WIPIs) to the nucleation site (Proikas-Cezanne *et al.*^2015^). Two ubiquitin-like conjugation systems, ATG12-ATG5 and the LC3 conjugation system, regulate incorporation of phosphatidylethanolamine (PE)-conjugated LC3 and promote elongation and closure of the autophagosome. During the final recycling step, another signaling phospholipid, PI(3,5)P2, mediates fusion of autophagosomes with lysosomes (Ferguson, Lenk and Meisler 2009). Mature autolysosomes ultimately digest the sequestered cargo and release amino acids and sugars into the cytoplasm of the cell (Berg *et al.*^1998^).

Selective autophagy, however, bypasses the nutrient-sensing kinases mTORC1 and AMPK and is restricted to specific targets in the cytoplasm, resulting in a localized autophagy response. Common substrates for selective autophagy are superfluous or damaged organelles, as well as large protein aggregates and long-lived proteins that are not accessible for the proteasome (reviewed in Levine, Mizushima and Virgin 2011). Moreover, there is a critical role for selective autophagy in the elimination of intracellular pathogens, a process also termed xenophagy (Fig. 1) (reviewed in Levine *et al.*^2011^). Cargo selectivity is mediated by autophagy receptors that couple specific substrates with the core autophagy machinery located on the pre-autophagic membrane. Polyubiquitin chains linked to the cargo serve as common substrate recognition signals and interact with the ubiquitin-binding domain of autophagy receptors. Autophagy receptors frequently possess a so-called LC3-interacting region (LIR) that mediates the crosstalk of the receptor with members of the LC3/GABARAP (gamma-aminobutyric acid receptor-associated protein) protein family (Birgisdottir, Lamark and Johansen 2013). Furthermore, receptor oligomerization amplifies interaction with downstream effectors. Although not mutually exclusive, distinct autophagy receptors are involved in various forms of selective autophagy (Table 1) (Xu *et al.*^2015^). Autophagy receptors related to xenophagy include nucleoporin 62/sequestosome 1 (p62/SQSTM1), neighbor of BRCA1 (NBR1), nuclear dot protein 52 (NDP52), optineurin (OPTN) and Tax1-binding protein 1 (TAX1BP1) (Thurston *et al.*^2009^; Zheng *et al.*^2009^; Mostowy *et al.*^2011^; Wild *et al.*^2011^; Tumbarello *et al.*^2015^; Verlhac *et al.*^2015^). Among the best-characterized receptors is p62/SQSTM1. Besides an established role in the clearance of intracellular pathogens, p62/SQSTM1 mediates degradation of different autophagy cargos, such as protein aggregates, mitochondria, peroxisomes and the midbody ring (Pankiv *et al.*^2007^; Geisler *et al.*^2010^). Complementary functions have been identified for the autophagy receptors OPTN and NBR1, which are involved in the clearance of mitochondria and peroxisomes, respectively (Kirkin *et al.*^2009^; Deosaran *et al.*^2013^). Functional redundancy of receptors extends the spectrum for the specific cargo. Many autophagy receptors act in concert to promote recognition and elimination of pathogens, further supporting the concept of an intracellular immune response.

**Table 1.** Summary of the receptors involved in selective autophagy.

Autophagy receptor	Ligands	LIR motif	References
p62/SQSTM1 (sequestosome 1)	LC3/GABARAP family, Ubiquitin, Keap1	YES	Pankiv *et al.* (2007); Komatsu *et al.* (2010); Lau *et al.* (2010)
NBR1	LC3/GABARAP family, Ubiquitin	YES	Kirkin *et al.* (2009); Wong *et al.* (2012); Deosaran *et al.* (2013)
NDP52/CALCOCO2	LC3C, Ubiquitin, Galectin-8, Myosin VI	YES	Thurston *et al.* (2009); Tumbarello *et al.* (2012)
TAX1BP1/CALCOCO3	Ubiquitin, Myosin VI	NO	Tumbarello *et al.* (2012, 2015)
OPT	LC3/GABARAP family, Ubiquitin, Myosin VI, p62	YES	Wild *et al.* (2011); Tumbarello *et al.* (2012)
NIX/BNIP3L	LC3/GABARAP family	YES	Schweers *et al.* (2007); Sandoval *et al.* (2008); Novak *et al.* (2010)
FUNDC1	LC3/GABARAP family, PGAM5, CK2	YES	Liu *et al.* (2012); Chen *et al.* (2014)


An alternative pathway to xenophagy is LC3-associated phagocytosis (LAP), which requires only some components of the autophagy machinery (Fig. 1). Indeed, LAP has emerged as an important mechanism in restricting the growth of different vacuole-enclosed microorganisms (Sanjuan *et al.*^2007^). During LAP, LC3 is incorporated directly into a pre-existing vacuole membrane and acts independently of the initiation complex ULK (Martinez *et al.*^2011^; Kim *et al.*^2013^). Since the substrate is already enclosed in a membrane, ubiquitination and receptor labeling of the cargo become unnecessary. Pivotal, however, are lipid modifications and the production of reactive oxygen species (ROS) to efficiently recruit the LC3 lipidation machinery (ATG5-ATG12-ATG16L complex and the LC3-conjugation system) to the target membrane (Huang *et al.*^2009^; Martinez *et al.*^2015^). Rubicon, the master regulator of LAP, activates the UVRAG-containing PI3KC3 complex, which generates PI3P to promote assembly of the NADPH oxidase (NOX) 2 complex. Rubicon is also engaged in the activation of the NOX2 complex to enhance the ROS response (Martinez *et al.*^2015^).

In *Plasmodium*-infected hepatocytes diverse autophagy pathways are activated during parasite development. While canonical autophagy serves as an important nutrient source during *Plasmodium* liver stage development, mechanisms related to either selective autophagy or LAP represent an intracellular immune response, which we summarize under the term *Plasmodium*-associated autophagy-related (PAAR) response (Fig. 1). Importantly, the PAAR response varies considerably in the different *Plasmodium* species investigated so far.

## PARASITE TRANSMISSION AND HEPATOCYTE INFECTION

Upon transmission by female *Anopheles* mosquitos, infectious sporozoites are deposited into the skin of their intermediate host during the blood meal (Frischknecht *et al.*^2004^). Sporozoites migrate through the host skin and actively enter a blood vessel (Amino *et al.*^2006^). Entering the host's blood circulation provides a fast shuttle to the parasite's primary destination, the liver. The immune-privileged status of the liver, its regenerative capacity as well as its high metabolic activity make it an optimal environment for the fast-growing parasite.

**Figure 3.**
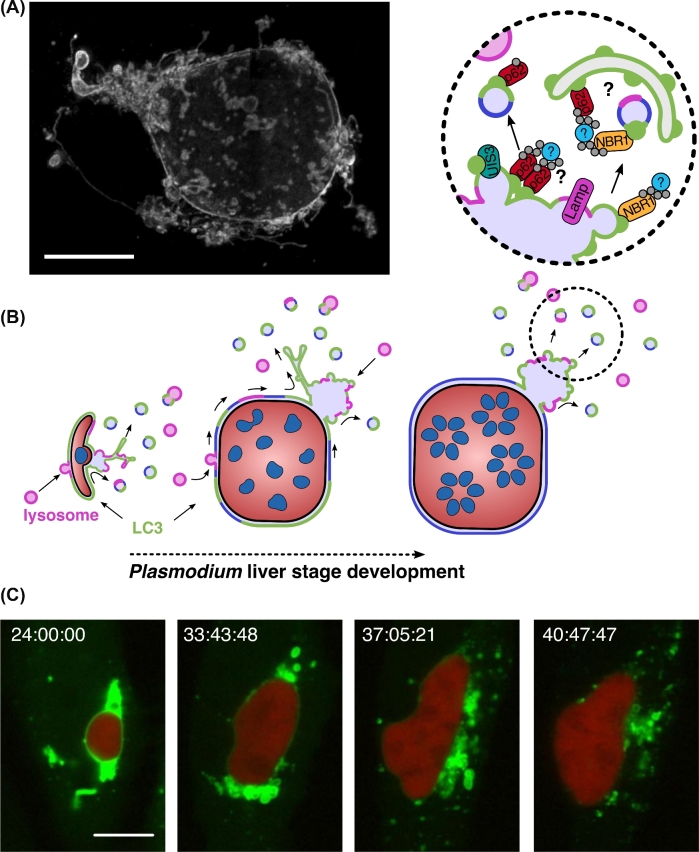
The parasitophorous vacuolar membrane (PVM) and its role during the shedding of host protein. (**A**) 3D architecture of the PVM and its connected tubovesicular network (TVN). Young *P. berghei* liver schizont (36 hpi) stained for the PVM protein exported protein 1 (EXP-1) was imaged by confocal laser scanning microscopy (3D-CLSM) with z-increments of 0.22 μm. Morphological features of the TVN represent highly branched tubular structures, large node-like clusters and vesicles. Scale bar, 10 μm. (**B**) Model of shedding mechanism involved in the removal of host autophagy (LC3) and lysosomal proteins from the PVM of *P. berghei* during liver stage development. PVM-associated host factors assemble in membrane patches at the PVM, accumulate and become trapped in the TVN, and are finally shed as vesicles into the host cytoplasm. Magnification of the TVN cluster highlights the potential link between the PAAR response and the nourishing capacity of the macroautophagy pathway. Adapted from Agop-Nersesian *et al.* (2017). (**C**) Long-term live microscopy of the shedding process. PMV-associated LC3 (green) is progressively removed from the developing *P. berghei* liver schizonts (24 hpi, red) into the cytoplasm of a HepG2 cell. Time stamp, h:min:s. Scale bar, 10 μm. Adapted from Prado *et al.* (2015).

During an active invasion process, the sporozoite invaginates the host cell plasma membrane to establish its replicative niche, the PV (Risco-Castillo *et al.*^2015^). One of the key determinants for recognition and productive invasion of hepatocytes is the 6-cysteine domain protein P36 on the sporozoite surface (Manzoni *et al.*^2017^). In analogy to merozoite invasion of red blood cells, the assembled moving junction is assumed to exclude host transmembrane proteins from entering the PVM during sporozoite invasion (Spielmann *et al.*^2012^). The parasite exports proteins to modify the molecular composition of the nascent PVM (Nyboer *et al.*^2018^). Protrusions formed from the PVM into the cytoplasm of the host cell are termed tubovesicular network (TVN). This architectural modification of the PVM is a continuous, membranous system. It is not only composed of dynamic tubular protrusions but also includes stationary cisternae-like clusters (Grützke *et al.*^2014^; Agop-Nersesian *et al.*^2017^), substantially expanding the surface area of the PVM (Fig. 3A). Within this customized and adaptable niche, the sporozoite differentiates from a trophozoite into a schizont, the replicative phase of the parasite. Upon completion of the asexual replication and formation of numerous infectious merozoites, the function of the vacuole becomes obsolete and the PVM disintegrates (Graewe *et al.*^2011^). PVM disintegration and merozoite release in the host cell cytoplasm result in the collapse of the host cell cytoskeleton and a controlled host cell death. As a consequence, the infected host cell detaches from neighboring cells in the liver parenchyma and forms merozoite-filled vesicles termed merosomes (Sturm *et al.*^2006^; Burda, Caldelari and Heussler 2017). Merosomes are transported through endothelia into a blood vessel and eventually detach from their mother cell to finally release infectious merozoites in the capillaries of the lungs (Sturm *et al.*^2006^; Baer *et al.*^2007^; Graewe *et al.*^2011^).

Interestingly, mutant parasites that cannot maintain a functional PVM are still able to infect hepatocytes, but very few of them develop into infectious merozoites. Genetic ablation of the two 6-Cys sporozoite proteins P52 and P36 resulted in liver stages that reside freely in the host cell cytoplasm as revealed by electron microscopy studies (Labaied *et al.*^2007^). Although some p52/p36-deficient parasites are able to complete liver development, their survival rate is dramatically reduced (Ploemen *et al.*^2012^; Annoura *et al.*^2014^). Genetically modified parasites with a defective PVM, induced by the loss of a single membrane protein like the upregulated in infective sporozoites (UIS) genes 3 or 4, are also efficiently eliminated in the cytoplasm of their host cells with very few parasites completing development (Mueller *et al.*2005a,b). Together, this strongly suggests that a functional PVM is necessary to protect the parasite from host cell responses while still allowing acquisition of nutrients for rapid parasite growth.

## THE PVM IS AN IMPORTANT INTERFACE BETWEEN THE PARASITE AND ITS HOST CELL

The PVM represents a natural barrier between the parasite and the hepatocyte's cytoplasm. Nevertheless, mammalian cells have developed sophisticated strategies to sense and eliminate vacuole-enclosed pathogens. While the endolysosomal and autophagy pathways are important recycling mechanisms that regulate cell homeostasis, host cells exploit this digestive capacity to control and eliminate intracellular pathogens. Interestingly, this bipolar function strongly influences *Plasmodium* development in the liver.

With the isolation from the host cytoplasm by the PVM, nutrient acquisition represents a challenge for the parasite. Despite its own metabolic capacity, the rapidly growing and replicating parasite additionally scavenges a variety of nutrients and metabolites from the host cell. *Plasmodium* liver stage parasites exhibit a strong dependency on exogenous lipid sources for successful development. During growth and replication, the parasite requires phospholipids to build its ER and plasma membrane as well as the extraparasitic TVN system (Grützke *et al.*^2014^; Kaiser *et al.*^2016^; Burda *et al.*^2017^). At the end of parasite development, when thousands of merozoites are formed in a very short time, there is again a strong demand of phospholipids to generate sufficient plasma membrane. Major sources might be the host cell and parasite ER. Interestingly, the PVM forms membrane contact sites with the parasite ER, which would enable direct translocation of lipids into the PVM (Kaiser *et al.*^2016^). On the other hand, the close association of host cell ER with the PVM (Bano *et al.*^2007^) could represent additional means of phospholipid scavenging for *Plasmodiumberghei*, but the contribution of these phospholipid sources to the plasma membrane of the newly forming merozoites requires further investigations.

*Plasmodium* seems to utilize multiple alternative scavenging pathways to compensate for the absence of certain lipid synthesis pathways. Indeed, many exported PVM-resident proteins interact directly with the lipid components of the host hepatocytes (Mikolajczak *et al.*^2007^; Petersen *et al.*^2017^; Sá e Cunha *et al.*^2017^). Exported protein 1 (EXP-1) was amongst the first parasite-derived proteins identified in the PVM (Simmons *et al.*^1987^; Kara *et al.*^1990^; Günther *et al.*^1991^). Only recently, however, we start to understand its molecular function. EXP-1 is expressed at different stages of the malaria life cycle. During the erythrocytic stage of the human malaria parasite *P. falciparum,* EXP-1 exhibits a glutathione S-transferase activity and is responsible for the detoxification of hematin (Lisewski *et al.*^2014^). In the hepatic stage, where the parasite does not rely on hemoglobin catabolism, the region of EXP-1 exposed to the host cytoplasm has been shown to directly interact with apolipoprotein H (ApoH). Moreover, internalization of ApoH may mediate the uptake of cholesterol (Sá e Cunha *et al.*^2017^). Like for cholesterol, rodent malaria parasites lack a *de novo* synthesis pathway for phosphatidylcholine (PC) (Déchamps *et al.*^2010^). Recently a member of the Fam-a protein family has been suggested to mediate transfer of PC from the host cell to the parasite (Fougère *et al.*^2016^). So far, in malaria research, multigene family members have been studied primarily in the context of antigen variation during the *P. falciparum* blood stage, where they are involved in the modification of the cell surface of the infected red blood cell (Spielmann and Gilberger 2015). The rodent model *P. berghei* encodes three distinct multigene families: Fam-a, Fam-b and PIR. While the function of Fam-b and PIR family proteins is unknown, Fam-a proteins appear to be involved in scavenging host phospholipids (Fougère *et al.*^2016^). Some *Pb*Fam-a proteins contain a characteristic steroidogenic acute regulatory-related lipid transfer (START) domain that is responsible for translocating lipids, such as fatty acids, ceramids or phospholipids, between membranes (Fougère *et al.*^2016^). The uptake and accumulation of host-derived PC is indeed essential for maintaining the membrane integrity as well as the protein composition of the PVM (Itoe *et al.*^2014^).

Among the best-characterized PVM-proteins are UIS 3 and 4, two members of the early transcribed membrane proteins (ETRAMPs). Both proteins are expressed already in sporozoites and are essential for early liver stage development (Mueller *et al*. 2005a,b). UIS3 directly interacts with the liver fatty acid binding protein (L-FABP), a lipid chaperone coordinating the lipid supply in hepatocytes and other tissue cells. Knockdown of L-FABP in HUH7 hepatoma cells impairs growth of *P. berghei*, suggesting that the parasite supplements its inherent fatty acid synthesis pathway in the apicoplast using this exogenous source (Mikolajczak *et al.*^2007^). The physiological function(s) of the UIS3-L-FABP interaction, however, remains to be elucidated. It is noteworthy that fatty acids are not restricted to cell metabolism. Many lipids can serve as secondary messengers to regulate different intracellular signaling pathways, such as modulation of inflammatory responses (Furuhashi and Hotamisligil 2008). It is therefore possible that lipid scavenging and modulation by the parasite might represent a strategy to manipulate the host's signaling cascades.

Although *Plasmodium* liver stages are known to interact with several host cell organelles (Bano *et al.*^2007^; Deschermeier *et al.*^2012^; Lopes da Silva *et al.*^2012^), the PVM-resident protein UIS4 represents the first identified parasite-derived molecular determinant responsible for host organelle interaction. In cells infected with UIS4-deficient parasites, recruitment of endolysosomal vesicles is strongly impaired and cholesterol accumulation in the PVM is affected (Petersen *et al.*^2017^). Besides UIS4-dependent recruitment of late endosomes (LE) and lysosomes, the PVM was found transiently modified with phosphatidylinositol-3-phosphate (PI3P) (Thieleke-Matos *et al.*^2014^). Differentially phosphorylated phosphatidylinositol (PI) species provide a unique membrane signature and orchestrate vesicular trafficking. By mimicking an early endosome ‘PI-code’, the PVM might regulate the fusion with host LE (Thieleke-Matos *et al.*^2014^). Moreover, pharmacological or genetic manipulation of the endolysosomal pathway results in developmental retardation of the parasite in the liver. The detection of endosomal content in the PV lumen endorses the fusogenic potential of the PVM. Therefore, infected hepatocytes retain an active endosomal and lysosomal machinery. In addition to cholesterol, endosomes could provide other secondary catabolic intermediates such as various glycoconjugates and amino acids (Lopes da Silva *et al.*^2012^; Thieleke-Matos *et al.*^2014^; Petersen *et al.*^2017^).

## INTERFERING WITH AUTOPHAGY MACHINERY HAS PHYSIOLOGICAL CONSEQUENCES FOR THE PARASITE

Canonical autophagy of the host cell represents an additional nutrient source for *Plasmodium* liver stage. Genetic manipulation of the host cell macroautophagy pathway revealed a general reduction in parasite growth (Prado *et al.*^2015^; Thieleke-Matos *et al.*^2016^; Wacker *et al.*^2017^). Since ATG5 is part of the LC3-lipidation pathway, ATG5-deficient cells are defective in initiating macroautophagy as well as alternative autophagy pathways such as selective autophagy or LAP. Infection of ATG5^flox/flox^ mice with *P. berghei* confirmed the physiological relevance of host cell autophagy for the parasite liver stage (Thieleke-Matos *et al.*^2016^). Although parasites in ATG5-deficient cells are subject to nutrient deprivation and thus develop slower, the overall survival rate of the parasite is significantly enhanced because the PAAR response also depend on ATG5 (Prado *et al.*^2015^; Wacker *et al.*^2017^) (Fig. 2). Supplementation of infected ATG5^–/–^ cells with amino acids, indeed, compensates for the parasite growth defect (Prado *et al.*^2015^).

In contrast, host cells deficient for ULK-associated protein FIP200 lack only the canonical autophagy pathway. As a consequence of the reduced nutrient supply, parasite growth is significantly impaired. Because PAAR responses are still functional in FIP200-deficient cells, parasite survival rates did not differ from those in wild-type cells (Wacker *et al.*^2017^). FIP200 and potentially other members of the ULK complex are dispensable for LC3 incorporation into the PVM. ATG5, however, which is essential for lipidation and thus membrane association of LC3, is required for LC3 recruitment to the PVM (Wacker *et al.*^2017^). Together, *P. berghei* liver stage parasites appear to benefit from nutrients supplied by canonical autophagy but are restricted by PAAR responses.

*In vivo* starvation experiments strongly support the antagonistic function of the different branches of host cell autophagy on the *Plasmodium* liver development. Mice starved during *Plasmodium* liver infection showed a more than 20-fold increase in parasite load compared to normal-fed infected mice. Part of the increased parasite burden was due to significantly larger parasites, probably supported by additional nutrients supplied by activated canonical autophagy. The major effect, however, was a drastically increased parasite survival rate upon host starvation (Prado *et al.*^2015^). An interesting hypothesis is that host cells might have limited supplies of molecules accessible for executing autophagy. If a starved cell becomes additionally infected, canonical and non-canonical autophagy pathways compete for common autophagy components. To survive starvation, the infected cell seems to prioritize canonical autophagy. According to this hypothesis, parasites could establish an infection more easily in starved cells, where they benefit from nutrients provided by canonical autophagy and they are not targeted by the PAAR response. The idea that each cell has a restricted number of autophagy proteins also has implications during co-infections of host cells, for example with viruses. In a recent study, co-infection of mice with adenovirus and *P. berghei* sporozoites drastically increased parasite liver load (Sá e Cunha *et al.*^2017^). Since adenoviruses are known to stimulate and exploit the host cell canonical autophagy machinery for their propagation (Hösel *et al.*^2017^), limited resources might again be the reason for the increased parasite load, similar to the effect observed upon starvation. It will now be interesting to decipher the molecular details of this complex interplay between distinct autophagy pathways during starvation and virus infection that lead to the increase in parasite survival.

## SENSING THE INTRACELLULAR INTRUDER FOR AUTOPHAGIC ELIMINATION

Intracellular pathogens eliminated by xenophagy are initially ubiquitinated and then recognized by autophagy receptors before they are labeled with the autophagy marker protein LC3 (Fig. 1). Although LC3 recruitment during *P. berghei* infection was observed, it does not follow a xenophagy response (Prado *et al.*^2015^; Schmuckli-Maurer *et al.*^2017^). In contrast to xenophagy, the receptor complex is recruited by LC3 to the PVM and thus follows an inverse order (Fig. 2). In LC3B-deficient host cells, both the autophagy receptors and ubiquitin are mainly lost from the PVM surface. Complementation of the LC3B-depleted host cells with a GFP-LC3 fusion protein rescues their PVM localization, confirming their LC3-dependent recruitment. While PVM localization of p62 is completely abolished in LC3 lipidation-deficient ATG5^–/–^ cells, the effect on NBR1 recruitment is relatively moderate. Further experiments are required to resolve the function of ubiquitin and whether accumulation of autophagy components at the PVM contributes to the anti-microbial response or is linked to nutrient scavenging induced by *Plasmodium* (Schmuckli-Maurer *et al.*^2017^). Since LC3 is not recruited via ubiquitination and subsequent receptor binding, what could be an alternative? Recently it has been shown that in *P. berghei*-infected hepatocytes, the parasite-exported PVM protein UIS3 can efficiently bind LC3 (Real *et al.*^2018^) possibly facilitating its incorporation into the PVM. How the parasite benefits from this recruitment is discussed in the next section about parasite evasion strategies from intracellular immune responses.

Different *Plasmodium* species deal differently with intracellular host cell immune responses. Whereas the PVM of invading *P. berghei* sporozoites is immediately labeled with LC3 (Grützke *et al.*^2014^; Prado *et al.*^2015^; Thieleke-Matos *et al.*^2016^), *P. vivax*-infected cells require additional IFN-γ stimulation to recognize and eliminate the parasite, using a mechanism closely related to LAP (Boonhok *et al.*^2016^). The fact that LC3 is directly incorporated into the PVM (Prado *et al.*^2015^; Boonhok *et al.*^2016^) supported this assumption. IFN-γ-mediated LAP response depends on Beclin 1, a member of the PI3KC3 core complex, and the ubiquitin-like conjugation system to promote LC3-PE incorporation and recruitment of acid vesicles to the PVM. Whether PI3P production requires other LAP-associated components of the PI3KC3 complex (UVRAG and Rubicon) and promotes NOX2-dependent ROS production remains an open question (Boonhok *et al.*^2016^).

Together, mechanisms mediating LC3 labeling of the PVM appear to vary between human and rodent *Plasmodium* species. The fact that host cells can react very individually on invasion by even closely related parasite species supports the concept of a flexible and diverse intracellular immune response.

## EVASION OF HOST AUTOPHAGY RESPONSES BY *PLASMODIUM*

As pointed out earlier, the parasite's ability to take advantage of the catabolic activity of the hepatocyte's autophagy machinery comes at a price. By retaining an active host autophagic and endosomal machinery, the parasite preserves the cytoprotective function of the hepatocyte. To successfully develop, the parasite needs to escape this response. Different *Plasmodium* species appear to have evolved alternative ways to deal with the digestive consequences of host cell autophagy.

In *P. berghei*, the PVM-resident protein UIS3 was recently shown to be a critical antagonist of the PAAR response. UIS3-deficient parasites arrest during liver-stage development and seem more susceptible to autophagy-mediated elimination (Mueller *et al.*2005b; Real *et al.*^2018^). Interestingly, when UIS3(-) parasites were allowed to develop in autophagy-deficient host cells, the mutants resumed blood stage infection comparable to wild-type parasites. *In vitro* experiments further revealed a direct interaction of LC3 with UIS3 through its non-canonical LIR motif. By shielding the LIR-binding site of LC3, UIS3 competes with other LIR motif-containing proteins such as p62 or Rab7 effector proteins (Real *et al.*^2018^). Since autophagy receptors are recruited to the PVM in an LC3-dependent manner (Schmuckli-Maurer *et al.*^2017^), UIS3 does not entirely abolish the interaction of LC3 with p62 and other autophagy receptors. The LIR-shielding function of UIS3 does also not explain why autophagy proteins are ultimately lost in late-stage schizonts from the PVM surface. Besides the UIS3-dependent sequestration of LC3, *Plasmodium* parasites use complementary evasion pathways.

A permanent association of LC3 with the PVM increases the likelihood for parasites to be eliminated (Prado *et al.*^2015^; Agop-Nersesian *et al.*^2017^). In order to remove PVM-associated autophagy proteins, the parasite induces membrane shedding from the PVM towards the TVN (Agop-Nersesian *et al.*^2017^). Expansion and plasticity of the TVN is especially pronounced during the proliferative phase of the liver stage parasite (Fig. 3) (Grützke *et al.*^2014^). To maintain membrane biogenesis, the PVM depends on a continuous lipid supply (Mikolajczak *et al.*^2007^; Itoe *et al.*^2014^) and a functional secretory system for constant export of proteins into the PVM. Based on the spatiotemporal redistribution of LC3 and ubiquitin, PVM-incorporated host cell proteins follow the TVN dynamics and accumulate in the cisternae-like clusters of the TVN. Different photobleaching and photoconversion approaches revealed that GFP-LC3 mobility was significantly reduced in the TVN compared to the PVM. The autophagy complex remains trapped in the TVN cluster, which serves as a primary disposal area. Although the molecular mechanism behind such a trap has not been resolved, it would be interesting to see if export of UIS3 might mediate the active transport of LC3 into the TVN. It is also conceivable that the convoluted architecture of the TVN could promote oligomerization of autophagy receptors, which remained in complex with PVM-associated LC3 (Fig. 3B). Aggregation of the TVN-bound receptor could additionally act as a decoy and exhaust the reservoir of autophagy proteins in the host cell cytoplasm (Agop-Nersesian *et al.*^2017^; Schmuckli-Maurer *et al.*^2017^). The controlled disposal of host cell proteins via the TVN has the advantage that potentially detrimental factors are contained in distance to the parasite's vulnerable replicative center. Moreover, vesicle-mediated shedding from the TVN guarantees spatial separation of autophagy proteins from the PVM (Fig. 3).

Since proliferating liver schizonts also depend on functional host cell endocytic pathways for nutrient uptake, *Plasmodium* does not interfere with endolysosomal biogenesis (Lopes da Silva *et al.*^2012^; Thieleke-Matos *et al.*^2014^; Petersen *et al.*^2017^). The antimicrobial action of autophagy on the other hand relies on fusion with the endolysosomal compartment. During *Plasmodium* infection, lysosome recruitment to the PVM can be a consequence of the PAAR response (Boonhok *et al.*^2016^; Zhao *et al.*^2016^) but occurs similarly in an autophagy-independent manner (Petersen *et al.*^2017^; Wacker *et al.*^2017^). How *Plasmodium* ultimately deals with the digestive capacity of the endolysosomal compartment is not fully understood. UIS4, however, could play a major role in the recruitment of LE and lysosomes (Petersen *et al*. 2017). Several other mechanisms have been proposed, which could function synergistically with UIS4. In order to reduce the potential of becoming exposed to hydrolytic enzymes, liver stage parasites might either restrict the overall number of fusion events or mediate selective fusion with less acidic LE (Lopes da Silva *et al.*^2012^). Additionally, *Plasmodium* liver stages regulate the permeability of their PVM. Indeed, molecules of up to 855 Da diffuse freely through the PV suggesting the presence of channels in the PVM (Bano *et al.*^2007^). Although the composition of such channels and their localization in the PVM are yet to be determined, the unselective passage of small molecules could counteract an acidification of the PV lumen. As a complementary mechanism, parasites might utilize protons for their own ATP synthesis or exploit them to import nutrients via cotransporters as it is known for bacteria and plants. For blood stage *P. falciparum* parasites, it has indeed been observed that ATPases localize to the plasma membrane of the parasite (Hayashi *et al.*^2000^). Notably, accumulation of autophagy-associated proteins and lysosomes around the PVM is especially pronounced during early phases of liver stage development (Lopes da Silva *et al.*^2012^; Prado *et al.*^2015^; Thieleke-Matos *et al.*^2016^; Zhao *et al.*^2016^; Agop-Nersesian *et al.*^2017^; Petersen *et al.*^2017^). It will now be important to investigate whether antiporter or proton-driven ATPases localize to the plasma membrane of early liver stage parasites.

In many ways, PVM shedding might assist in controlling the overall load of host lysosomes at the PVM surface. During liver stage development of *P. berghei*, PVM-associated lysosomal markers display the same spatial and temporal dynamics as LC3 (Prado *et al.*^2015^). Analogous to the LC3-receptor complex, lysosomes might first become trapped in the TVN and then be subsequently shuffled back into the hepatocyte cytoplasm via vesicular shedding. The finding that, despite constant interaction of LE/lysosomes with the PVM, only a limited concentration of lysosomal markers are found associated with the PVM and TVN (Lopes da Silva *et al.*^2012^; Petersen *et al.*^2017^) supports such a scenario. In addition, macroautophagy may recycle the TVN-shed vesicles and thus contribute to the nourishment of the parasite (Fig. 3B). A shedding-dependent evasion strategy, coupled to recycling of vesicle contents, would support the notion of the multifaceted impact of host autophagy on the parasite development (Agop-Nersesian *et al.*^2017^).

For the rodent malaria parasite *P. yoelii,* it has been suggested that the parasite survives in LC3-positive autophagosome-like vacuoles. Although escape mechanisms like PVM shedding have not been studied, it was discussed that *P. yoelii* prevents the maturation into an autolysosome (Zhao *et al.*^2016^).

In contrast to the evasion strategy observed in *P. berghei*, *P. vivax* liver stage parasites escape the host's autophagy machinery by avoiding selective recognition of the parasite. LC3 labeling of the PVM of *P. vivax* is induced by the effector cytokine IFN-γ (Boonhok *et al.*^2016^). Parasites targeted for elimination by IFN-γ−mediated LAP generally fail to evade the intracellular immune response. A unique feature of *P. vivax* is its ability to form dormant stages in the liver, the hypnozoites. It is reasonable to speculate that *P. vivax* prevents initiation of an autophagic response by interfering with the IFN-γ signaling pathway, in order to facilitate hypnozoite differentiation and persistence.

The direct comparison of different evasion mechanisms of diverged human and rodent *Plasmodium* species remains difficult, in particular since the duration of liver stage development is considerably longer in human *Plasmodium* species compared to rodent species. Nevertheless, it is important to understand the evolutionary pressure driving microbial defense mechanisms. Since the hypnozoites of *P. vivax* are metabolically not very active, shedding-mediated removal of host autophagy proteins may be a less efficient measure to restrict lysosome targeting the parasite. Future research will hopefully reveal how hypnozoites deal with the host cell autophagy response.

## CONCLUSIONS

The PVM is the major interface for the parasite in sensing and manipulating its host cell environment. Besides offering physical protection for the replicating parasite, the PVM simultaneously remains permeable for large numbers of metabolites. The multifunctional role of the PVM is reflected by its complex architecture and compartmentalization that results in the generation of an extensive TVN. In many aspects, the PVM/TVN play central roles in counteracting the intracellular immune response of the hepatocyte. One of the most interesting tasks will be to dissect the molecular divergence between the different *Plasmodium* species that induce distinct hepatocyte responses. Especially in the context of *P. vivax,* whose dormant stages present a major challenge for the development of effective antimalarials, our understanding of the parasite's evasion mechanisms is still rudimental. Future challenges will be to decipher more molecular details of the parasite's evasion strategies and use them as a basis for new therapeutics.
